# Can the goals of rehabilitation and disability be reconciled? A discussion paper

**DOI:** 10.3389/fresc.2026.1768125

**Published:** 2026-05-07

**Authors:** Jerome Bickenbach, Birgit Prodinger, Alessandro Giustini, Gerold Stucki

**Affiliations:** 1 Swiss Paraplegic Research, Nottwil, Switzerland; 2 Chair of Inclusive Health Care, Faculty of Medicine, University of Augsburg, Germany; 3 Rehabilitation Research Institute, San Raffaele, Rome, Italy; 4 European College of Physical and Rehabilitation Medicine (UEMS-PRM), Brussels, Belgium; 5 Faculty of Health Sciences and Medicine, University of Lucerne, Lucerne, Switzerland; 6 Center for Rehabilitation in Global Health Systems, WHO Collaborating Center, University of Lucerne, Lucerne, Switzerland

**Keywords:** activism, disability, functioning, ICF, inclusion, rehabilitation

## Abstract

The objective of this short note is to ask How can we make sense of and reconcile these two societal trends? Our aim is not to provide our own definitive answer, supported by argument and evidence, but to interest the research community to weigh in and either reject the premise that there is a tension, or propose approaches to reconciliation. With that in mind, we begin by providing two, very general, reasons for thinking there is a tension – mindful that there is diverse academic literature here that we cannot adequately summarize – and then making a suggestion about one possible approach to reconciliation.

## Introduction

Because of population ageing, a gradual increase in the prevalence of chronic, noncommunicable diseases, improved health care and medical and technical advances in prevention and early diagnosis, the future of health care needs is changing (1, 2). Increasingly, people will live longer but with more chronic limitations in functioning – that is, with more disability. As a health care strategy, rehabilitation focuses on the individual patient, the disease or injury as well as mobility, sensory, communication, cognitive or other impairments that impact daily life. The experience of disability is also determined by the person's interaction with the environment, the presence or absence of accommodation and support to enhance inclusion and full participation in society. With rehabilitation, the person is at the focus of the intervention, while with human rights it is society itself that is the actor as it is responsible for removing systemic barriers and facilitating inclusion. There are, therefore, at least two fundamental social strategies to respond disability: strengthening and increasing access to rehabilitation and using a human rights approach as a guide for laws, policies and practices to ensure inclusion of people with disability.

These two social strategies were located in different sectors: health and social sectors in the case of rehabilitation and legal and policy sectors for social integration. Historically, the two approaches have evolved and matured differently, responding to different socio-economic drivers: rehabilitation was primarily a response to the needs of returning wounded soldiers after the First World War and an expansion of the spa tradition in Europe (3). The impetus to focus on physical obstacles to mobility and communication, stereotypical misunderstanding of the capacities of persons experiencing disability, and outright bias, prejudice and discrimination came primarily from a social movement of advocates for people with disabilities (4).

More recently, these two societal strategies have been officially expressed, by the World Health Organization’s Resolution on Rehabilitation: Strengthening Rehabilitation in Health Systems (5) and earlier by the United Nations Convention on the Rights of Persons with Disabilities (6). The WHO Resolution was the product of a multi-year initiative and call for action Rehabilitation 2030. This sets out an agenda to strengthen rehabilitation in national health systems, in order to serve the estimated 2.4 billion people worldwide who could benefit from rehabilitation, and especially underserved populations (7). The UN Convention was the result of decades-long, bottom-up social movement of disability advocates who argued that people with disabilities as a historically disadvantaged minority group denied basic human rights that prevent their full inclusion and participation in society.

The objective of this short note is to ask How can we make sense of and reconcile these two societal trends? Our aim is not to provide our own definitive answer, supported by argument and evidence, but to interest the research community to weigh in and either reject the premise that there is a tension, or propose approaches to reconciliation. With that in mind, we begin by providing two, very general, reasons for thinking there is a tension – mindful that there is diverse academic literature here that we cannot adequately summarize – and then making a suggestion about one possible approach to reconciliation.

## A tension between rehabilitation and disability inclusion?

While there is an extensive academic literature on the relationship between rehabilition and disability politics, especially within disability studies, there are arguably two, very general, sources of this tension, especially expressed in policy. The first involves a common distinction made in social policy between universal and targeted programs. Universal programs provide benefits and services to every individual in a population, while targeted programs select candidates for support within that population based on an eligibility screening in terms of a demographic, behavioral or other characteristics of the individual. While universal programs will provide benefits even for those groups who may not need them, in order to be universal in coverage, targeted programs have to sacrifice universal coverage for targeted benefits. In short, this distinction is relevant to the contrast between rehabilitation and inclusion: Since society has an obligation to respond to the health and rehabilitation needs of everyone, access should be universal. On the other hand, the aim of social inclusion can, logically, only be satisfied by targeting those who have been excluded. The tension between rehabilitation and disability inclusion appears just to be a special case of the intrinsic tension between universal and targeted societal responses is salient.

Another source of a potential tension between universalistic rehabilitation and targeted disability inclusion policies involves the term ‘disability’ itself (8). The term can be used either to refer to a universal human experience or as an identifier of a disadvantaged minority, social group. It is this latter sense that is part of a wider conceptual and political approach called the ’social model of disability’, which asserts that features of the social and political environment create, and are primarily responsible for social exclusion and the denial of basic human rights. By contrast, in the first sense of the term, disability is rooted in biomedical impairments that are universal and unavoidable biological facts of human life, but which, in different environments, may result in limitations in social participation and exclusion.

## The tension and reaching out

There has been a history of animosity among disability advocates against the ‘medicalization’ of disability by, among others, rehabilitation professionals (9, 10). But it is important to recognize that, however legitimate the general concern about over-medicalizing disability among health professionals has been, in the case of rehabilitation, the story is more nuanced. Not only have rehabilitation providers sought to embrace a wider, social justice conception of their roles. In many ways the development of Community Based Rehabilitation (CBR) was an attempt to extend the range of rehabilitation to include community-based disability advocacy (11), and the prominent role of rehabilitaiton in the integration of assistive technology provision in the social sector and inclusive empoloyment policies, illustrate the sensitivity of the rehabilitation profession to the need to enhance, and practically facilitate social inclusion for persons with disabilities. Moreover, some rehabilitation professions have explicitly argued that professional role includes but extends beyond patients and their families to society at large and issues of social justice (12–14). The largest international professional organization, Rehabilitation International, was, for example, highly engaged in the development and implementation of the UN Convention. These and other social initiatives, originating and implemented by the rehabilitation community, are grounds for being optimistic that reconciliation is a real possibility.

## The case for reconciliation

Is there a case to be made for reconciliation? The motivation for this brief paper is primarily to invite contributions to an on-going debate about whether, and if so, how a reconciliation might be achieved. Without in any way pre-empting this debate, but perhaps in providing some hints at a resolution, a few points can be made: Firstly, social policy specialists have become uncomfortable with a strict universal/targeted distinction. This side of the question can be best expressed in the language of functioning {found in the World Health Organization's *International Classification of Functioning, Disability and Health* [ICF (13)]}. In the ICF, disability is a limitation in functioning in one or many domains, experienced by a person with an underlying health condition or impairment as a problem of performing some action, activity or social role in the actual physical, attitudinal, and social environment in which the person acts. Since impairments exist on a spectrum of severity, it is reasonable to conclude that disability too is a matter of ‘more or less’, not ‘yes or no’. Epidemiologically, the bulk of impairment and therefore disability experienced across the population will be mild or moderate in severity. So, while severe disability, or disability created from adverse social conditions such as stigma or discrimination, are only experienced by a minority, as a general matter and across the spectrum of severity disability is universally experienced.

Importantly, the ICF operationally recognizes that the limitations a person may experience go beyond those directly linked to the health condition, and extend to the full range of human activities, simple to complex. These limitations, moreover, a created by features of the individual's environment, both physical and human-built environment and the vastly more complex social, political, economic and cultural environment in which the person is engaged in complex activities and social roles, from engaging in work, education, and family life to participating in civic and community life.

The ICF recognizes, in short, that the social and political environment can be a determinate of the limits in social inclusion that are experienced. But, as the severity of impairment and disability is continuous, society's response must be tailored to the actual needs of people experiencing disability. The response may be directed at the intrinsic limitations associated with impairments, or more broader to environmental facilitators (such as assistive technology) or social and political change that addresses the environment determinations of the limitations of disability. In ICF terms, the aim of the social response to disability is to optimize functioning in the person's actual environment in all relevant areas of life, where functioning is understood broadly to comprise not merely bodily function, but also functioning at the level of activities and social participation. Sometimes this requires biomedical intervention to address the underlying disease or injury, or to moderate the associated impairments; sometimes it requires changes in the person's environment to optimize their performance and increase full inclusion and participation.

In this sense, rehabilitation is an example of an institutional social response to disability that addresses the underlying phenomenon of function; indeed, rehabilitation can be defined as the health strategy that optimizes functioning at the person-level (14). Rehabilitation accomplishes this by combining functioning interventions addressing the patient's own targets and life goals and environment interventions that, together support persons in the translation of intrinsic health capacity into real-life performance in their own environments, including by providing assistive technology or environmental modifications to home, school of employment environments.

Rehabilitation is a performance-enhancing social response, but obviously not the only one. Social activism and the human rights approach in favor of people with disabilities is another form of social response, and has the same goal – optimizing performance for persons with disabilities to enhance participation and inclusion. Inasmuch as people who are characterized – either socially or by self-identification – as people with disabilities tend to have severe impairments and more extensive needs, they are a minority group who are more widely socially disadvantaged. Advocacy for this minority will tend to focus on the highest societal level in terms of the legal, social, economic and political changes required to achieve inclusion. But this societal goal is arguably just another version of optimizing functioning in social participation. Of course, disability activism is multi-dimensional and involves not merely the high-level political and legal change, but also a wide range of community organizing and grassroots mobilization for accessibility, the removal barriers and the consequent enhancing of autonomy, independence and self-determination (15). But despite many differences in approach, methodology and short- and long-term, both the health and social strategy represented by rehabilitation and the human rights approach to social and political advocacy for people with disability, aim at optimizing functioning. Having a similar ultimate goal of inclusion and full participation, the differences between rehabilitation and disability advocacy may not be as saliant as their underlying consiliance.

## Conclusion

How can we reconcile the person-focused rehabilitation and disability as universal human phenomena, with the societally-focused, human rights approach of disability advocacy for the minority group of people with disabilities? Although we have suggested that, using the ICF's notion of functioning to characterize a common goal that might reconcile rehabilitation and disability activism, it might be argued that rehabilitation cannot shed its associated with a medicalized approach, or that the social dynamics of disability activism means that the social goal of inclusion cannot plausibly be reduced to that of optimal functioning. The challenge of reconciling the common goals of rehabilitation and the drive for social inclusion and full participation is important enough to engage both community. So, we encourage researchers to take up this challenge and we invite submissions to the Research Topic “Can the goals of rehabilitation and disability be reconciled?” to debate this issue.

## Data Availability

The original contributions presented in the study are included in the article/supplementary material, further inquiries can be directed to the corresponding author.

## References

[1] Chatterji S Byles J Cutler D Seeman T Verdes E. Health, functioning and disability in older adults–present status and future implications. Lancet. (2015) 385(9967):563–75. 10.1016/S0140-6736(14)61462-8 PMID: 25468158 PMCID: PMC4882096. 25468158 PMC4882096

[2] United Nations, Department of Economic and Social Affairs, Population Division. World Population Ageing 2024. New York: United Nations (2024). Available online at: https://www.un.org/development/desa/pd/sites/www.un.org.development.desa.pd/files/undesa_pd_2024_wpp_2024_advance_unedited_0.pdf (Accessed April 28, 2026).

[3] Caracheo A Bickenbach J Stucki G. The emergence of the rehabilitative strategy: the driving forces in the United States of America. Am J Phys Med Rehabil. (2018) 97(3):222–8. 10.1097/PHM.0000000000000864 PMID: 29189210. 29189210

[4] Bickenbach JE. Ethics, law, and policy. In: Albrecht GL, Series Editor. The SAGE Reference Series on Disability: Key Issues and Future Directions. Vol. 4. Thousand Oaks, CA: SAGE Publications (2012). p. 360.

[5] World Health Organization. Strengthening Rehabilitation in Health Systems. WHA76.6 Resolution, Adopted 30 May 2023. Geneva: World Health Organization (2023). Available online at: https://apps.who.int/gb/ebwha/pdf_files/WHA76/A76_R6-en.pdf (Accessed April 28, 2026).

[6] United Nations General Assembly. Convention on the Rights of Persons with Disabilities. A/RES/61/106; Adopted 13 December 2006; Entered into Force 3 May 2008. New York: United Nations (2006). Available online at: http://www.un.org/disabilities/convention/conventionfull.shtml (Accessed April 28, 2026).

[7] Cieza A Causey K Kamenov K Hanson SW Chatterji S Vos T. Global estimates of the need for rehabilitation based on the Global Burden of Disease study 2019: a systematic analysis for the Global Burden of Disease Study 2019. Lancet. (2021) 396(10267):2006–17. 10.1016/S0140-6736(20)32340-0 33275908 PMC7811204

[8] Bickenbach J Rubinelli S Stucki G. Being a person with disabilities or experiencing disability: two perspectives on the social response to disability. J Rehabil Med. (2017) 49(7):543–9. 10.2340/16501977-2251 28661547

[9] Albrecht GL. The Disability Business: Rehabilitation in America. Newbury Park, CA: SAGE Publications (1992). p. 327.

[10] Schick A Asch A Wasserman D. Disability: III. Theories of. In: Jennings B, editor. Bioethics. 4th edn. Farmington Hills, MI: Macmillan Reference (2014). p. 867–74.

[11] World Health Organization UNESCO ILO, International Disability and Development Consortium. Community-Based Rehabilitation: CBR Guidelines. Geneva: World Health Organization (2010). Available online at: https://www.who.int/publications/i/item/9789241548052 (Accessed April 28, 2026). 26290927

[12] Alston RJ Harley DA Middleton R. The role of rehabilitation in achieving social justice for minorities with disabilities. J Vocat Rehabil. (2006) 24(3):129–36. 10.3233/JVR-2006-00327

[13] Bailliard AL Dallman AR Carroll A Lee BD Szendrey S. Doing occupational justice: a central dimension of everyday occupational therapy practice. Can J Occup Ther. (2020) 87(2):144–52. 10.1177/0008417419898930 PMID: 31964168. 31964168

[14] McGrath RL Shephard S Chen YT. The need for transformational change in social justice–informed physiotherapy. Physiother Can. (2024) 76(3):241–3. 10.3138/ptc-2022-0108 40959154 PMC12392831

[15] Scotch R. From good will to civil rights: Transforming federal disability policy. Temple University Press (2009).

